# Case Report of Acute Pancreatitis Associated With Combination Treatment of Dulaglutide and Glipizide

**DOI:** 10.7759/cureus.20938

**Published:** 2022-01-04

**Authors:** Oyedotun Babajide, Nabin K C, Isaac Solaimanzadeh, Zewge Shiferaw-Deribe

**Affiliations:** 1 Internal Medicine, Interfaith Medical Center, Brooklyn, USA; 2 Internal Medicine/Endocrinology, Interfaith Medical Center, Brooklyn, USA

**Keywords:** medication-induced pancreatitis, diabetes mellitus type 2, glipizide, dulaglutide, acute pancreatitis

## Abstract

The objective of this report is to discuss a case of drug-induced acute pancreatitis in a patient on a combination of dulaglutide and glipizide. The patient was a 61-year-old African American male with a past medical history of diabetes mellitus type 2 and essential hypertension, who was admitted for acute pancreatitis after presenting with upper abdominal pain. He was initially on glipizide but dulaglutide was added to improve control. The patient was a social drinker and an ex-cigarette smoker. He had serum lipase greater than 900 U/L, serum alcohol was negative, and abdominal computed tomography reported significant pancreatic edema consistent with acute pancreatitis but without features of necrotizing pancreatitis and no evidence of cholelithiasis or choledocholithiasis. His clinical state deteriorated after being complicated by paralytic ileus. He was managed conservatively, improved clinically, and was discharged home.

Seeing that the incidence of pancreatitis is higher in patients with diabetes when compared to non-diabetics, it is important to counsel and monitor patients for risk factors of pancreatitis including medications. In the absence of other common causes in this case and considering the temporal relationship between presentation and the addition of dulaglutide to ongoing glipizide regimen, the combination of both drugs may have induced acute pancreatitis.

## Introduction

Acute pancreatitis is an acute inflammation of the pancreas postulated to be mediated by the breakdown of the pancreatic acini by pancreatic enzymes that can be triggered by a host of various conditions. The exact mechanism leading to this is unknown, but several risk factors have been identified in the pathogenesis of acute pancreatitis. Common causes implicated are gallstones, alcoholism, and hypertriglyceridemia, and other minor causes include medications. The incidence of acute pancreatitis in the United States ranges from 4.9 to 35 per 100,000 population [1]. In the United States, gallstones account for 40-70% of cases [2] and alcohol is responsible for about 25-35% [3]; on the contrary, acute pancreatitis caused by medications account for less than 5% and usually takes a milder course [4]. It is also important to note that the incidence of acute pancreatitis is higher in patients with type 2 diabetes when compared with non-diabetics [5], and studying added risk by medications is very important. Most of the evidence supporting this is based on retrospective studies and case reports. The lack of adequate prospective studies and underreporting could be the reason for a low incidence of drug-induced pancreatitis.

Common drugs known to cause acute pancreatitis include azathioprine, mesalazine/sulfasalazine, didanosine, oestrogens, and diuretics like furosemide and hydrochlorothiazide. Case reports remain an important tool in evaluating the possibility of expanding the list of medications implicated in drug-induced pancreatitis. This case report focuses on the possible role of dulaglutide, a glucagon-like peptide-1 receptor agonist, in combination with glipizide, a sulphonylurea, in inducing pancreatitis.

Drug-induced pancreatitis is diagnosed similarly to other common forms of acute pancreatitis using the revised Atlanta Classification for Acute Pancreatitis. This requires two or more of the following: abdominal pain suggestive of pancreatitis, elevated serum amylase or lipase greater than three times the upper limit of normal, or findings characteristic of pancreatitis on abdominal imaging. It is important to note that a proper history is very crucial in diagnosis as well as finding possible causes of pancreatitis.

Management is mostly supportive with resuscitation, intravenous hydration, bowel rest, analgesia, and of course stopping the suspected offending medication in the case of drug-induced pancreatitis.

## Case presentation

A 61-year-old African American male with a past medical history of diabetes mellitus type 2 and essential hypertension was admitted to the step-down unit with complaints of sudden upper abdominal pain for two days prior to the hospital admission. The patient reported developing severe pain in the right upper quadrant about an hour following his lunch. The pain was sharp, rated 8 out of 10 in severity, and radiated to the back. It was associated with nausea and repeated vomiting. Vomitus was non-bloody and contained food particles. The review of other systems was essentially normal. Home medications included 10 mg of glipizide daily and 0.75 mg of dulaglutide weekly. He was previously on metformin and glipizide for years, but metformin was switched to dulaglutide about six months before presentation. The patient was a social drinker with about two to three bottles of beer a week, with his last drink being three days before the presentation. He is an ex-cigarette smoker and his surgical and family histories were non-contributory.

**Figure 1:**
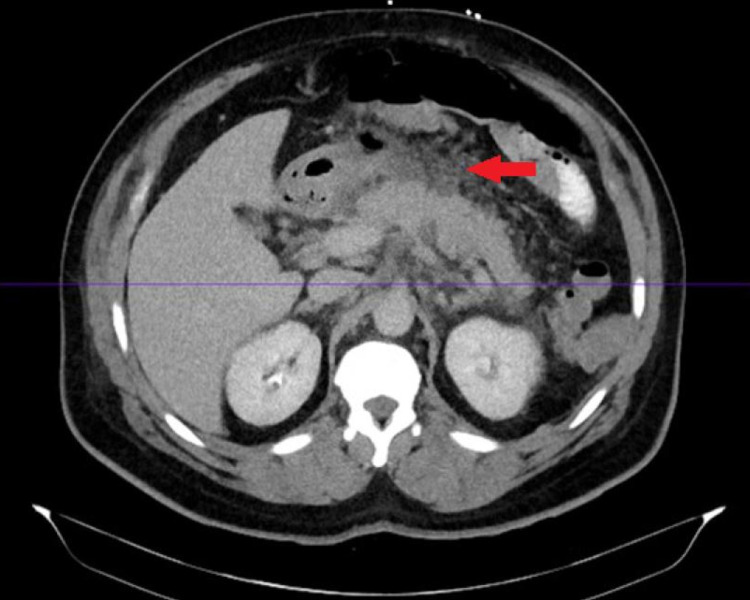
Computed tomography of the abdomen with intravenous contrast. Red arrow showing increased peripancreatic fat stranding, fluid, and possible early phlegmon suggestive of acute pancreatitis. An organized abscess or other fluid collection is not seen.

The temperature on presentation was 98.2°F, heart rate was 82 beats per minute, blood pressure was 195/80 mmHg, and oxygen saturation was 96% on room air. On physical examination, he was found to have moderate tenderness over the right upper quadrant and epigastrium with guarding. The examination was negative for rebound tenderness and bowel sounds were normoactive in all quadrants. The rest of the physical examination was non-significant. He had leukocytosis with a white blood cell (WBC) count of 21.8 x 109/L and significant left shift (neutrophils 95%). Lipase was more than 900 U/L and serum creatinine level was 1.28 mg/dL. His serum calcium level was 8.9 mg/dL, sodium level was 139 mEq/L, bicarbonate level was 30 mEq/L, and he had an anion gap of 13. Serum bilirubin, transaminases, and venous blood gas were normal and serum ketones and alcohol were negative. Triglycerides were 54 mg/dl, total cholesterol level was 161 mg/dl, and low-density lipoprotein (LDL) level was 97 mg/dl. Ranson’s score on admission was 3. Computed tomography (CT) of the abdomen with contrast (Figure 1) reported significant pancreatic edema consistent with acute pancreatitis but without features of necrotizing pancreatitis and was also negative for cholelithiasis, choledocholithiasis, or pancreatic duct calcifications. Chest X-ray revealed bilaterally increased density in both lower lung zones.

The patient got admitted for acute pancreatitis. He was managed conservatively with adequate intravenous hydration with nothing by mouth and pain controlled with parenteral morphine as needed. Diabetes and hypertension were controlled with insulin and amlodipine, respectively. On day two of admission, he developed abdominal distension with hypoactive bowel sounds. Abdominal X-ray and repeat CT abdomen showed features of dilated small bowels with no signs of intestinal obstruction. The patient was upgraded to the intensive care unit. Supportive management was continued, and the stomach was decompressed with nasogastric suction as recommended by the surgical team. His abdominal pain and distension gradually improved on day four of admission and his diet was advanced as tolerated. Serum lipase gradually declined to 209 and 114 (Table 1). WBC trended down to 11 x 109/L; serum calcium, sodium, potassium, blood urea nitrogen, and creatinine remained unremarkable. The patient was discharged on metformin with a hemoglobin A1c level of 7.7 and with instructions to follow up with his primary care physician. On follow-up five months after discharge, he is on long-acting insulin in addition to metformin and has had no further episodes of acute pancreatitis since stopping dulaglutide and glipizide.

**Table 1:** Significant laboratory results.

Labs	Day 1	Day 2	Day 3	Day 4	Day 5	Day 6
Lipase (U/L)	>900 H	209 H	114 H	230 H	297 H	
White blood cell count	21.8 x 1000	25.3 x 1000	19.9 x 1000	18.4 x 1000	17.8x 1000	11 x 1000
Serum calcium	9.2 mg/dl	8.7 mg/dl	8.3 mg/dl	8.8 mg/dl	9 mg/dl	9.4 mg/dl
Serum creatinine	0.83 mg/dl	0.87 mg/dl	0.82 mg/dl	0.84 mg/dl	0.81 mg/dl	0.83 mg/dl
Serum bicarbonate	24 mEq/L	28 mEq/L	26 mEq/L	27 mEq/L	27 mEq/L	28 mEq/L
Triglycerides	54 mg/dl					

## Discussion

Dulaglutide acts like an incretin mimetic on glucagon-like peptide-1 (GLP-1) receptors; it increases the secretion of insulin by the pancreas in response to glucose, decreases the secretion of glucagon, and can also delay gastric emptying [6]. Dulaglutide is effective in improving glycemic control and lowering hemoglobin A1c [7]. Glipizide, a sulfonylurea, increases insulin stimulation by the pancreatic beta cells. It also increases the responsiveness of beta cells and tissue sensitivity to insulin [8].

Since the approval of dulaglutide by the Food and Drug Administration (FDA) in 2014, it continues to gain traction in the number of yearly prescriptions with an estimated 4.2 million prescriptions in 2018 [9]. When it comes to adverse reactions, the gastrointestinal system is the most affected, with symptoms such as nausea, vomiting, abdominal pain, and diarrhea being reported [7]. Elevated amylase and lipase but within normal limits have also been reported with the use of dulaglutide [7]. Pancreatitis as an adverse reaction was also reported during clinical trials of dulaglutide, with 1.4 cases per 1,000 patient-years in patients exposed to dulaglutide as compared to 0.88 cases per 1,000 patient-years in non-incretin control groups [10]. In a systematic review and meta-analysis of 60 studies on treatment with GLP-1 receptor agonists or dipeptidyl peptidase-4 (DPP-4) inhibitors in adults with type 2 diabetes mellitus compared with placebo, the incidence of pancreatitis was found to be low in patients using incretins when compared to placebo [11] but exenatide, a GLP-1 agonist, has been shown to have an increased odds ratio for reported pancreatitis of up to six-fold when compared to other therapies [12].

Glipizide was approved by the FDA in 1984 and has been widely used ever since. The most common adverse reaction of glipizide is hypoglycemia but gastrointestinal symptoms like nausea and diarrhea have also been reported. Glipizide is not known as a common medication to cause drug-induced pancreatitis; however, when compared to DPP-4 inhibitors or metformin, it may be associated with an increased risk of pancreatitis [13].

In the management of diabetes, combining anti-diabetic medications to achieve better glycemic control is commonplace as seen in our patient that was on glipizide and dulaglutide. Both medications have gastrointestinal adverse reactions but neither has been proven to cause acute pancreatitis; however, not much is known about if there is a synergistic effect concerning adverse reactions when both medications are used together especially considering that both the GLP-1 agonist, exenatide and glipizide, might have increased risk of pancreatitis as compared to other therapies [12].

Another point to consider is that dulaglutide can cause adverse reactions that are similar to symptoms of acute pancreatitis, and it also causes an increase in amylase and lipase. A lot of these symptoms and the elevation in the enzymes are mild and hence rarely reported; one could hypothesize that it plays a more important role in causing pancreatitis as due to the usually mild course of drug-induced pancreatitis, most cases are underreported. Cases could also be misclassified under other etiologies when a proper history is failed to be obtained. The patient then runs the risk of resuming the medication, which could lead to another episode. Although most cases of drug-induced pancreatitis are mild, our index case had to be managed in the critical care unit due to paralytic ileus as a result of the illness.

## Conclusions

Seeing that the incidence of pancreatitis is higher in patients with diabetes when compared to non-diabetics, it is important to counsel and monitor patients for risk factors of pancreatitis including medications. In the absence of other common causes in this case and considering the temporal relationship between presentation and the addition of dulaglutide to ongoing glipizide regimen, the combination of both drugs may have induced acute pancreatitis.

Cases of adverse drug reactions should be reported to the necessary authorities by filling an event form on the US FDA website. More cases should be reported if suspected and further clinical trials especially on drug combinations such as dulaglutide and other anti-diabetic medications may be necessary to study possible adverse reactions associated with the combinations.
